# Working towards full eradication of lipid-driven cardiovascular risk?

**DOI:** 10.1007/s12471-021-01600-8

**Published:** 2021-07-19

**Authors:** N. S. Nurmohamed, E. S. G. Stroes

**Affiliations:** 1 Department of Vascular Medicine, Amsterdam UMC, University of Amsterdam, Amsterdam, The Netherlands; 2 Department of Cardiology, Amsterdam UMC, Vrije Universiteit, Amsterdam, The Netherlands

**Keywords:** Cardiovascular disease risk, Low-density lipoprotein cholesterol, Lipids, Apolipoprotein, Lipoprotein(a), Triglycerides

## Abstract

Lipid-driven cardiovascular disease (CVD) risk is caused by atherogenic apolipoprotein B (apoB) particles containing low-density lipoprotein cholesterol (LDL-C), triglycerides and lipoprotein(a) [Lp(a)] and resembles a large and modifiable proportion of the total CVD risk. While a surplus of novel lipid-lowering therapies has been developed in recent years, management of lipid-driven CVD risk in the Netherlands remains suboptimal. To lower LDL‑C levels, statins, ezetimibe and proprotein convertase subtilisin/kexin type 9 inhibiting antibodies are the current standard of therapy. With the approval of bempedoic acid and the silencing RNA inclisiran, therapeutic options are expanding continuously. Although the use of triglyceride-lowering therapies remains a matter of debate, post hoc analyses consistently show a benefit in subsets of patients with high triglyceride or low high-density lipoprotein cholesterol levels. Pemafibrate and novel apoC-III could be efficacious options when approved for clinical use. Lp(a)-lowering therapies such as pelacarsen are under clinical investigation, offering a potent Lp(a)-lowering effect. If proven effective in reducing cardiovascular endpoints, Lp(a) lowering holds promise to be the third axis of effective lipid-lowering therapies. Using these three components of lipid-lowering treatment, the contribution of apoB-containing lipid particles to the CVD risk may be fully eradicated in the next decade.

## Introduction

An atherogenic lipid profile is one of the most important risk factors for atherosclerotic cardiovascular disease (CVD) [1]. Lipid-driven risk represents a substantial proportion (approximately 17%) of the population attributable risk for CVD [2]. First, the largest proportion of the lipid-driven CVD risk is caused by low-density lipoprotein cholesterol (LDL-C) containing apolipoprotein B (apoB) lipoproteins. About one third of the Western population has dyslipidaemia, defined as an elevation of LDL-C-containing apoB lipoproteins [1, 3]. Second, in a significant proportion of patients the lipid-driven risk is caused by elevation of triglyceride- and apoB-containing lipoproteins. Almost equal to the prevalence of elevated LDL‑C, 31% of the Western population is hallmarked by a mild-to-moderate hypertriglyceridaemia [4]. Third, an estimated 20% of the world population has elevated lipoprotein(a) [Lp(a)] levels above 50 mg/dl [5].

While all three risk factors constitute highly prevalent conditions, treatment remains suboptimal and further steps are needed to reduce the impact of lipids on the CVD risk. Thus, 31% of Dutch secondary prevention and 77% of primary prevention patients are not achieving their guideline-advised LDL‑C targets [6]. Triglyceride-lowering therapies have not been very effective in reducing the CVD risk, while the triglyceride risk burden is increasing due to the pandemic of obesity and diabetes. Finally, approximately 1% of patients have extremely high Lp(a) levels exceeding 180 mg/dl, which places them at a familial hypercholesterolaemia (FH)-like risk elevation [7], while therapies significantly lowering Lp(a) levels are not yet in clinical use.

Fortunately, many novel lipid-lowering therapies targeting LDL‑C, triglycerides and Lp(a) have emerged over the last decade. The purpose of this review is to discuss current and novel therapies for treating the lipid-driven CVD risk and to support the clinician in the choice between different targets and therapies.

## LDL-C/apoB lowering

Retention and accumulation of apoB—mostly LDL—particles are mandatory for the initiation of atherosclerotic plaque formation. In all major epidemiological studies, the amount of LDL‑C and apoB is causally related to the CVD risk. Randomised trials in over 180,000 patients have shown that every 1 mmol/l reduction of LDL‑C is associated with a 19–23% decrease in CVD risk [8]. Genetic evidence has substantiated that apoB provides the best estimate of the CVD risk [9]. This proves the concept that the CVD risk is determined by the number of apoB particles, rather than the nature of the particle carrying the cholesterol. Mendelian randomisation studies show that lifetime genetic inactivation of pathways targeted by lipid-lowering drugs [3-hydroxy-3-methylglutaryl coenzyme reductase (HMGCR), Niemann-Pick C1-like 1 (NPC1L1) and proprotein convertase subtilisin-kexin type 9 (PCSK9)] results in comparable relative risk reductions per mmol/l LDL‑C or amount of apoB lowering [10, 11]. Taking the duration of exposure into account, an individual’s LDL-C-driven CVD risk consists of the cumulative exposure to apoB particles, comparable to the number of pack-years cigarette smoking [12].

### Current options: statins, ezetimibe and PCSK9 inhibitors

The development of the first LDL-C-lowering therapies started in the middle of the past century and led to the development of drugs such as niacin, fibrates and bile acid sequestrants, which did not consistently lower the CVD risk and were hampered by various side effects [13]. Statins were the first LDL-C-lowering therapies to effectively reduce the CVD risk. They lower plasma LDL‑C by upregulating hepatic LDL-receptor expression through inhibition of the rate-limiting step of cholesterol biosynthesis, hydroxymethylglutaryl (HMG)-CoA reductase. High-dosed statin therapy leads to a 30–50% reduction in LDL‑C, while less intensive statin regimens yield a 20–30% LDL‑C reduction [8, 14, 15]. Over the years, statins have proven to be very safe. Although adverse effects are relatively common in open-label surveys, randomised controlled trials show a low adverse event rate [16]. Observed adverse effects include new-onset diabetes (10–20 cases per 10,000 patient-years) and myopathy (1 case per 10,000 patient-years) [16]. However, the clinical prevalence of statin intolerance remains high and 7–29% of patients experience statin-associated muscle symptoms, resulting in a major drawback in statin prescription and adherence [17].

In 2015, the IMPROVE-IT trial provided evidence that ezetimibe was also effective in reducing major adverse cardiovascular events (MACE) when given on top of statins [hazard ratio (HR) 0.94, 95% confidence interval (CI) 0.89–0.99, *p* = 0.016] after an acute coronary syndrome [18]. Ezetimibe reduced LDL‑C by an additional 18–22% by targeting the NPC1L1 protein, thereby reducing the intestinal absorption of cholesterol [19, 20]. The results of IMPROVE-IT were confirmed by Mendelian evidence showing that reduction of the CVD risk is comparable per mmol/l genetic LDL‑C lowering by *NPC1L1* and *HMGCR* polymorphisms [11]. In the IMPROVE-IT trial, ezetimibe was generally well tolerated and without major adverse effects. However, ezetimibe may result in (albeit mostly mild) gastrointestinal side effects.

PCSK9 inhibitors prevent extracellular binding and degradation of LDL receptors by inhibition of the PCSK9 protein and are the most recent addition to the lipid-lowering armamentarium. Currently available PCSK9 inhibitors are monoclonal antibodies which are administered subcutaneously twice monthly: evolocumab and alirocumab. They were proven effective in the FOURIER and ODYSSEY OUTCOMES trials, respectively, comprising a total of 46,488 patients, showing an additional reduction of 59–62% in LDL‑C when given in addition to statin therapy [21, 22]. Both studies led to an additional 15% MACE reduction after a mean follow-up of 2.2 and 2.8 years respectively. There were no major safety issues with either drug, and adverse event rates were similar to those with placebo, except for a higher occurrence of injection site reactions with PCKS9 inhibitors. In addition, treatment with PCSK9 inhibitors can result in flu-like symptoms in a small proportion of patients.

### Bempedoic acid

In 2020, both the European Medicines Agency (EMA) and the United States Food and Drug Association (FDA) approved bempedoic acid for use in patients with statin intolerance or patients not reaching LDL‑C targets using statin therapy [23, 24]. Bempedoic acid can be prescribed as a daily oral drug and is metabolised in the liver to a coenzyme A (CoA) thioester form, which targets adenosine triphosphate citrate lyase (ACL) upstream of HMG-CoA reductase, resulting in upregulation of LDL receptors and a reduction of plasma LDL‑C [25]. Its mechanism was supported by a Mendelian randomisation study in 654,783 subjects, showing variants in the ACL gene, *ACLY*, resulted in a similar CVD risk reduction per mmol/l LDL‑C lowering compared to variants in the statin gene, *HMGCR *[26]. In clinical trials, bempedoic acid resulted in a 17–23% reduction of LDL‑C when given on top of maximally tolerated statin therapy or in statin-intolerant patients [27–29]. Importantly, the incidence of muscle-related adverse events was comparable to that of placebo in these trials. Bempedoic acid has an acceptable safety profile, however did result in an increase of blood uric acid levels and an increased risk of gout (1.2% vs 0.3%, *p* = 0.03) in the phase 3 CLEAR Harmony trial (NCT02666664). The larger phase 3 CLEAR Outcomes trial (*n* = 14,014) will provide a more detailed profile for CV efficacy and safety (NCT02993406).

### Inhibition of PCSK9: inclisiran

In addition to PCSK9-inhibiting monoclonal antibodies, other promising therapies targeting PCSK9 have emerged. Inclisiran is a subcutaneously administered small interfering RNA (siRNA) [30], providing intracellular, hepatocyte-specific PCSK9 inhibition by silencing PCSK9 messenger RNA (mRNA) translation [31]. Inclisiran is long-acting following incorporation into the RNA-induced silencing complex leading to sustained LDL‑C reductions, which allows twice-yearly administration. Phase 3 trials have corroborated a consistent 50% reduction of LDL‑C in CVD patients, CVD risk equivalent patients and FH patients [32, 33]. Adverse event rates were generally similar between groups, with the exception of (mild) injection-site reactions occurring more frequently in the treatment groups. The ORION‑4 trial will provide long-term data on CV efficacy and safety (NCT03705234). On 11 December 2020, inclisiran was approved by the EMA for the treatment of adults with hypercholesterolaemia or mixed dyslipidaemia, with two doses a year.

### ANGPTL3 inhibition

The liver-expressed, secreted protein angiopoietin-like protein 3 (ANGPTL3) inhibits lipoprotein lipase (LPL), preventing hydrolysis of triglycerides in the peripheral capillaries. In addition, ANGPTL3 also lowers apoB and LDL‑C by increasing clearance of LDL particles and reducing hepatic very-low-density lipoprotein (VLDL) secretion [34, 35]. Importantly, its mechanisms of action are independent of the low-density lipoprotein receptor (LDLR) pathway. Genetic studies have shown that *ANGPTL3* loss-of-function mutations result in a hypobetalipoproteinaemia phenotype, hallmarked by decreased levels of triglycerides, LDL‑C and high-density lipoprotein cholesterol (HDL-C) [34, 36]. Inhibition of ANGPTL3 with evinacumab was recently shown to be effective in reducing LDL‑C levels by 49% in a phase 3 trial with 65 FH patients already maximally treated with lipid-lowering therapies [37]. In this study, triglycerides were lowered by 50% and another phase 3 trial will evaluate its efficacy in patients with severe hypertriglyceridaemia (NCT03452228). There have been no major safety issues in the clinical studies with evinacumab to date, and the FDA has designated evinacumab a breakthrough status, paving the way for efficient approval.

In addition to evinacumab, multiple other drugs targeting ANGPTL3 are currently in clinical trials. IONIS-ANGPTL3‑L_Rx_, an antisense oligonucleotide (ASO) targeting ANGPTL3, showed dose-dependent LDL‑C and triglyceride reductions in its phase 1 study [38]. Its phase 2 study is currently underway in patients with type 2 diabetes, hypertriglyceridaemia and fatty liver disease (NCT03371355). ARO-ANG3 is an siRNA agent targeting ANGPTL3, and preliminary data also showed moderate dose-dependent reductions in LDL‑C and triglycerides.

### Clinical use of LDL-C-lowering agents

In all patients with a high CVD risk and LDL‑C levels above their target according to the European Society of Cardiology (ESC)/European Atherosclerosis Society (ESA) guidelines [7], potent statin therapy is the first-choice treatment. In all patients not achieving target LDL‑C levels, ezetimibe should be added to potent statin therapy. If elevated LDL‑C levels are present in a very-high-risk patient, direct initiation of combination therapy of potent statin and ezetimibe can be considered. In cases of very high CVD risk and residual LDL‑C burden, PCSK9 inhibition can be considered to further reduce LDL‑C. Due to the higher costs of PCSK9-inhibiting antibodies, reimbursement is limited to patients following myocardial infarction *and *(1) recurrent myocardial infarction; (2) diabetes mellitus; (3) documented statin intolerance; or (4) in patients with FH not reaching LDL‑C target levels despite statin/ezetimibe combination therapy. Pending reimbursement, bempedoic acid can also be considered to reduce LDL‑C in patients not reaching the LDL‑C target or in patients with statin intolerance. Finally, evinacumab is a promising additional option for patients with (homozygous) FH, since it lowers LDL‑C independently of the LDL receptor pathway.

## Triglyceride lowering

Hypertriglyceridaemia is present in approximately 30% of the Western population, but its burden continues to grow due to the increasing prevalence of obesity and diabetes. Obesity is associated with an atherogenic lipid profile hallmarked by high plasma triglyceride levels [39]. Both population and genetic studies have provided evidence for a causal relationship between triglycerides and CVD [40]. By means of Mendelian randomisation it was shown that triglyceride-lowering variants in the *LPL* gene achieved a CVD risk reduction equal to that of LDL-C-lowering variants in the *LDLR *gene per unit of apoB lowering [41]. This supports the concept that the CVD risk reduction is dependent on the reduction of apoB particles, and not on the magnitude of LDL‑C or triglyceride lowering.

### Current options: fibrates and omega-3 fatty acids

The dependency on apoB reduction may partly explain the failure of large fibrate trials to show a solid reduction in CVD, since the achieved apoB lowering using fibrate therapy is minimal [42]. However, in post hoc analyses, fibrates were found to reduce the CVD risk in patients with elevated triglyceride and/or low HDL‑C levels at baseline [43–45]. Pemafibrate is a novel fibrate which selectively targets peroxisome proliferator-activated receptor (PPAR)-alpha. Given orally once daily, pemafibrate appears to have a favourable adverse effect profile and is more potent, offering both LDL‑C as well as apoB lowering [46, 47]. The ongoing PROMINENT trial evaluating pemafibrate in more than 10,000 type 2 diabetes patients with mixed dyslipidaemia will show whether there is a place for triglyceride-lowering regimens in future CVD prevention (NCT03071692).

Another point of debate is the use of omega‑3 fatty acids. The supposed mechanism of action of daily dosed omega‑3 fatty acids is largely unknown and pleiotropic [48]. After failure of almost all omega‑3 fatty acid trials, REDUCE-IT was the first study to show a significant MACE reduction [49]. In 8179 patients, high-dose eicosapentaenoic acid (EPA) significantly reduced the primary endpoint in the intervention group compared to placebo (HR 0.75, 95% CI 0.68–0.83, *p* < 0.001). More recently, the STRENGTH trial in 13,078 patients, which evaluated high-dose EPA in combination with docosahexaenoic acid, was terminated early due to its futility [50]. Whether the discrepancy between these studies in part reflects the poorly chosen placebo (mineral oil) in the REDUCE-IT trial or is the result of differences between the two formulations remains a matter of debate. Importantly, the CVD benefit in the REDUCE-IT trial is unlikely to be solely attributable to the modest triglyceride lowering by high-dose EPA [48].

### ANGPTL3 inhibition

For patients with a residual triglyceride risk, or a combined LDL-C/triglyceride risk, inhibition of ANGPTL3 is an interesting therapeutic option that lowers both LDL‑C and triglycerides. It is discussed in the LDL-C-/apoB-lowering section.

### Targeting of apoC-III

ApoC-III (apoC-III) is an apolipoprotein mainly carried by triglyceride-rich lipoproteins, such as chylomicrons and VLDLs. apoC-III prevents hydrolysis of triglycerides by LPL, for example [51]. In a genetic study, carriers of loss-of-function mutations in *APOC3* had an HR of 0.59 (95% CI 0.41–0.86, *p* = 0.007) and 0.64 (95% CI 0.41–0.99, *p* = 0.04) for ischaemic vascular disease and ischaemic heart disease respectively [52]. Volanesorsen, an ASO, was the first apoC-III inhibitor reaching clinical trials, and is given as a subcutaneous formulation twice monthly. Treatment with volanesorsen effectively reduces plasma triglyceride levels in familial chylomicronaemia syndrome and hypertriglyceridaemia by approximately 70–80% [53]. However, volanesorsen is hampered by the high incidence of adverse effects, including thrombocytopenia and injection-site reactions [54], precluding use in large patient populations. Recently, a N-acetyl galactosamine-conjugated (GalNAc_3_) ASO targeting apoC-III (AKCEA-APOCIII‑L_Rx_) was tested in a phase 1/2a trial [55]. The GalNAc_3_ formulation enables specific delivery to the liver, increasing potency while minimising systemic exposure. Indeed, the trial showed potent reductions in triglycerides up to 92%, and administration was without major adverse effects.

### Clinical use of triglyceride-lowering agents

Since the CVD benefit of current triglyceride-lowering agents is controversial, they should not be prescribed routinely. Fibrates can be considered only in very-high-risk patients with persisting high triglyceride levels whose risk cannot be reduced with LDL‑C lowering. EPA can also be considered in patients with a high CVD risk who have elevated triglyceride levels, although EPA has not been approved by the EMA to date. Highly potent pemafibrate, if shown to be effective and tolerable, could prove an interesting option in patients with an atherogenic lipid profile with high triglyceride levels, such as diabetes patients. AKCEA-APOCIII‑L_Rx_, the GalNAc_3_ ASO targeting apoC-III, is a promising new therapy to reduce triglycerides and could, if reasonably priced, be a future therapy choice in the patient with a high CVD risk and high triglyceride levels. In patients with a combined ‘refractory’ LDL-C/triglyceride risk, ANGPTL3 inhibition could kill two birds with one stone.

## Lowering of Lp(a)

Lp(a) is an LDL-like particle with its apoB covalently bound to an apo(a) tail. Plasma levels are genetically determined for more than 90% [56]. Elevated plasma Lp(a) has been established as a causal risk factor for both CVD and calcific aortic valve disease in population and genome-wide association studies [57, 58]. Further genetic evidence shows that gain-of-function mutations in the *LPA *gene increase the CVD risk, while loss-of-function mutations lead to a reduction of the CVD risk [59]. The prevalence of elevated Lp(a) is high; 1.4 billion people worldwide have an Lp(a) level above the threshold of 50 mg/dl [5]. In addition, approximately 1% have an Lp(a) level above 180 mg/dl, which puts them at an FH-like risk of CVD [7]. Unfortunately, a large proportion of patients remain unidentified due to unawareness among physicians and the previous lack of an effective Lp(a)-lowering treatment. The latter is about to change: multiple Lp(a)-lowering therapies are currently in clinical trials.

### Current options: PCSK9 inhibitors

Current options to lower Lp(a) are scarce. Conversely, statins are known to slightly increase Lp(a) levels up to 10–20% [60]. The only therapy in use which is known to lower Lp(a) is PCSK9 inhibition. In addition to their effective LDL‑C reductions, PCSK9 inhibitors reduce Lp(a) levels by a median of 21–26% [61]. It was estimated, based on Mendelian randomisation studies, that relatively large absolute Lp(a) reductions between 50 and 100 mg/dl are necessary to achieve a CVD risk reduction equal to that of 1 mmol/l LDL‑C reduction [62, 63]. However, a recent analysis of the ODYSSEY OUTCOMES trial has shown that PCSK9 therapy could be of use in patients with very high Lp(a) levels. It was observed that Lp(a) reduction independently lowered the CVD risk (2.5% risk reduction per 5 mg/dl Lp(a) lowering by alirocumab). The CVD risk reduction achieved with alirocumab in patients in the highest Lp(a) quartile was for 39% attributed to Lp(a) reduction. This suggests that a subgroup of patients with a high CVD risk and a high Lp(a) level could benefit from PCSK9 inhibition.

### Pelacarsen (TQJ230)

Pelacarsen (ASO) is the first Lp(a)-lowering drug in a phase 3 trial and is administered as a subcutaneous injection once monthly. With its GalNAc_3_ conjugation, potency is increased while systemic side effects are reduced. Pelacarsen has shown dose-dependent decreases in Lp(a) up to 92% in its phase 2 trials [64, 65]. In these trials, there were no major safety issues and pelacarsen was generally well tolerated, with the exception of more injection-site reactions in the pelacarsen group than with placebo. The phase 3 trial of pelacarsen, Lp(a) HORIZON, is currently recruiting 7680 patients with established CVD and is expected to be completed in 2024 (NCT04023552).

### siRNA: AMG890 and SLN360

Two investigational siRNA therapies targeting Lp(a) mRNA are currently being clinically evaluated. First, AMG890 (olpasiran) is an siRNA,the phase 1 study of which has recently finished, showing dose-dependent reductions of more than 90% without any safety concerns [66]. These reductions persisted for 3–6 months in the highest doses. Its phase 2 trial in patients with elevated Lp(a) is currently underway (NCT04270760). Second, SLN360 is another siRNA drug which has shown potent Lp(a) reduction without any adverse or off-target effects in animal studies;its phase 1 study will be starting soon (NCT04606602).

### Clinical use of Lp(a)-lowering agents

If proven effective for lowering of the CVD risk, and approved by regulatory agencies, Lp(a)-lowering treatments could provide the clinician with the last tool to abolish residual apoB-mediated lipid risk. These therapies should be prescribed in patients with a high CVD risk (secondary prevention) with elevated Lp(a) levels (> 50 mg/dl). In patients with extremely high levels > 180 mg/dl and hence a markedly increased lifetime CVD risk, Lp(a) lowering should also be considered. Following the most recent ESC/EAS dyslipidaemia guidelines, every at-risk individual should have their Lp(a) levels measured at least once in their lifetime. It is especially important to measure Lp(a) when measuring other lipids, since Lp(a) cholesterol and Lp(a)-apoB are measured in the total concentration of LDL‑C or apoB respectively. Therefore, in patients with very high Lp(a) levels, the measured LDL‑C and apoB could be ‘falsely’ elevated and actually reflect elevated Lp(a)-C and Lp(a)-apoB. These patients would benefit much more from Lp(a) lowering than from LDL-C/apoB lowering. This problem could be solved by routinely reporting LDL‑C levels corrected for Lp(a)-C [67].

## Conclusions

**Fig. 1:**
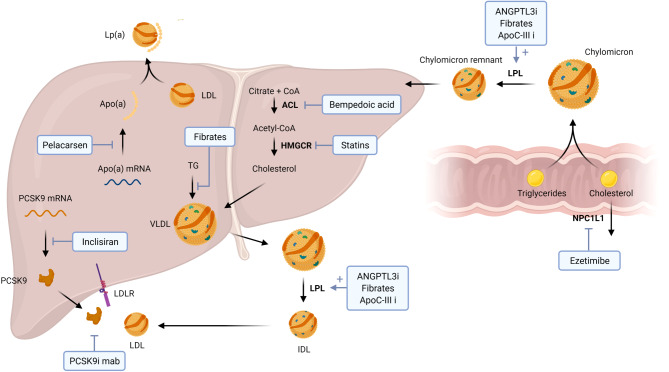
Mechanisms of action of the discussed lipid-lowering therapies. Pelacarsen inhibits apolipoprotein(a) (*apo[a]*). Inclisiran prevents translation of proprotein convertase subtilisin kexin type 9 (*PCSK9*) mRNA. PCSK9i (inhibiting) monoclonal antibodies (*mab*) inhibit PCSK9 binding to low-density lipoprotein receptor (*LDLR*). Fibrates mainly prevent synthesis of triglycerides (*TG*) and very-low-density lipoprotein (*VLDL*) production in the liver. Bempedoic acid prevents cholesterol synthesis by inhibition of adenosine triphosphate citrate lyase (*ACL*). Statins block 3‑hydroxy-3-methylglutaryl coenzyme reductase (*HMGCR*). Angiopoetin-like 3 protein inhibitors (*ANGPTL3i*), fibrates and apoC-III inhibitors (*apoC-IIIi*) enhance lipoprotein lipase (*LPL*) function. Ezetimibe targets Niemann-Pick C1-like 1 protein (*NPC1L1*), inhibiting transport of sterols into enterocytes. Created with BioRender (BioRender.com). *CoA* coenzyme A, *IDL* intermediate-density lipoprotein, *Lp(a)* lipoprotein(a)

There is an abundance of lipid-lowering therapies under development in addition to the currently available regimens (Fig. 1). The vast majority of these agents are safe with minimal side effects, which compares favourably to agents targeting other pathways such as coagulation (bleeding risk) and inflammation (infection risk). In each individual patient, a personalised treatment dependent on their CVD risk and respective lipid profile should be configured. In patients with a high LDL‑C risk, bempedoic acid and the twice-yearly dosed inclisiran are novel options on top of guideline-advised statins, ezetimibe and PCSK9-inhibiting antibodies. When high triglyceride levels are the main cause of the residual CVD risk, options to lower this risk are currently limited, but pemafibrate and novel apoC-III inhibitors provide a future perspective. For patients with high Lp(a) levels, pelacarsen is the first of several therapies which potently lower Lp(a), and can be prescribed if proven safe and effective in reducing MACE. In summary, the contribution of apoB-containing lipid particles to the CVD risk can be fully eradicated within the next decade in a safe and highly effective manner.
